# Villains or heroes? The raison d'être of viruses

**DOI:** 10.1002/cti2.1114

**Published:** 2020-02-19

**Authors:** Tokiko Watanabe, Yoshihiro Kawaoka

**Affiliations:** ^1^ Division of Virology Department of Microbiology and Immunology Institute of Medical Science University of Tokyo Tokyo Japan; ^2^ Department of Pathobiological Sciences School of Veterinary Medicine University of Wisconsin‐Madison Madison WI USA; ^3^ Department of Special Pathogens International Research Center for Infectious Diseases Institute of Medical Science University of Tokyo Tokyo Japan

**Keywords:** neo‐virology, viral purpose, viral roles in the ecosystem

## Abstract

The relationship between humans and viruses has a long history. Since the first identification of viruses in the 19th century, we have considered them to be ‘pathogens’ and have studied their mechanisms of replication and pathogenicity to combat the diseases that they cause. However, the relationships between hosts and viruses are various and virus infections do not necessarily cause diseases in their hosts. Rather, recent studies have shown that viral infections sometimes have beneficial effects on the biological functions and/or evolution of hosts. Here, we provide some insight into the positive side of viruses.

## Graphical abstract

Traditionally, viruses have been considered to be ‘pathogens’ that cause diseases in their hosts. In this review, we discuss recent studies that shed light on previously unrecognised beneficial roles of viruses in nature.

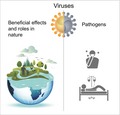

## Introduction

Viruses, which consist of nucleic acid encased in a protein shell, are parasites of host organisms. The term ‘virus’ comes from the Latin word ‘venom’, which means poison, because a virus is generally considered to be a causative agent like a poison that causes infectious diseases. These tiny, living entities have considerable import, because they can cause substantial damage to humans and non‐human animals and other living organisms. The relationship between humankind and viruses has a long history. For example, the earliest evidence of smallpox was found in 3000‐year‐old Egyptian mummies, who had smallpox‐like eruptions on their skins.^1^ The overall mortality rate of smallpox was around 30%,^2^ making it one of the most feared infectious diseases. In 1918–1919, during World War I, influenza A virus caused the Spanish flu pandemic, resulting in infection of approximately 500 million people and more than 20–40 million death worldwide.^3^

Since the initial isolation of viruses in the 19th century, scientists have identified and characterised a wide variety of viruses, and the field of virology has progressed remarkably since then, enabling us to combat the frequently deadly effects of these viruses. One of the greatest achievements is the complete eradication of smallpox. Although smallpox was once rampant in the world, vaccination of the entire population has eradicated this disease.^1^ Similarly, the poliovirus vaccine has significantly reduced the incidence of poliomyelitis.^4^ Despite the progress of virology, we still have many unconquered viral diseases and we are confronted with the problem of emerging infectious diseases, which are caused by newly identified species or strains. For example, Ebola virus disease and acquired immunodeficiency syndrome emerged in 1976 and 1981, respectively,^5^, ^6^, ^7^, ^8^, ^9^ and more recently, severe acute respiratory syndrome (SARS), highly pathogenic avian influenza viruses and Middle East respiratory syndrome (MERS) have appeared in human society.^10^, ^11^, ^12^, ^13^, ^14^, ^15^ Therefore, it is important to continue studying the mechanisms of viral replication and pathogenicity.

Yet, these negative aspects of viruses do not tell the whole story since the relationships between hosts and viruses are multitudinous, and virus infections do not necessarily lead to disease symptoms in hosts. Rather, recent studies suggest that there are viruses that are beneficial to the biological functions and/or evolution of their hosts. Recently, we established a research consortium, designated as ‘Neo‐virology’, which is supported by Grants‐in‐Aid for Scientific Research on Innovative Areas from the Ministry of Education, Culture, Science, Sports, and Technology (MEXT) of Japan. In this consortium, we define a virus as a component of the global ecosystem. Our aim was to elucidate the roles of viruses in host organisms and the global ecosystem, in contrast to traditional virology research, which tends to focus on pathogenic viruses that cause diseases in their hosts. This research project is expected to develop into an important scientific field that examines the interactions between the global ecosystem and viruses. In this brief review, we give some insights into the positive side of viruses.

## Beneficial effects of viral infection on mammalian hosts

In traditional virology, most viruses found in humans are considered to be pathogenic to their hosts; however, recent studies have shown that there are some viruses that have symbiotic relationships with their hosts and do not cause disease. Infection with one virus may protect the host from a superinfection with another pathogen. Barton *et al*.^16^ demonstrated that latent infection with the herpesviruses murine gammaherpesvirus 68 or murine cytomegalovirus, which are genetically related to the human pathogens Epstein‐Barr virus and human cytomegalovirus, respectively, led to cross‐protection in mice. Infection with these viruses induced prolonged production of the antiviral cytokine interferon‐gamma and systemic activation of macrophages that protected the mice from subsequent bacterial infections with either *Listeria monocytogenes* or *Yersinia pestis*.^16^ Moreover, it has been reported that superinfection with hepatitis A virus suppressed hepatitis C virus replication in patients with chronic hepatitis C in at least two cases,^17^ and infection with human cytomegalovirus (HCMV) suppressed superinfection with HIV‐1 *in vitro* as a result of the downregulation of the expression of CCR5, a co‐receptor for HIV‐1, induced by the HCMV infection.^18^

Some viruses also have beneficial effects with respect to non‐infectious diseases. Epidemiologic studies suggest that virus infections in childhood might confer protection against some cancers later in life. For example, the risk of chronic lymphoid leukaemia in subjects who had measles in childhood is relatively low,^19^ and mumps infection in childhood might protect against the development of ovarian cancer in adults.^20^ However, infection with oncoviruses is known to increase the risk of development of some cancers (e.g. cervical cancer and liver cancer induced by the human papillomavirus and hepatitis B virus/hepatitis C virus infection, respectively).^21^ Such information is important when considering strategies for cancer immunotherapy and/or vaccination campaigns. In addition, the infection of non‐obese diabetic mice with lymphocytic choriomeningitis virus prevented the infected mice from developing autoimmune disease and subsequent type I diabetes (insulin‐dependent diabetes mellitus).^22^, ^23^ Chronic viral infection of mice with murine cytomegalovirus (CMV) increased epithelial turnover and wound repair via antiviral cytokine type I interferons (IFNs),^24^ but CMV infection can promote cancer malignancy; this phenomenon is known as ‘oncomodulation’.^25^, ^26^

Recent metagenomic studies have revealed that virus infection sometimes confers benefits including the regulation of microbiota in the gut. Bacteriophages are abundant in the gut and are thought to modulate the gut microbiota by infecting specific bacterial populations. Accordingly, potential therapeutic applications of bacteriophages in humans (e.g. control of antibiotic‐resistant bacteria, stabilisation of healthy gut microbes) have been considered.^27^, ^28^

Therefore, the elucidation of the symbiotic effects of viruses on the physiological functions and immune responses of their hosts, as well as clarification of the functional mechanisms involved, will lead to an understanding of the essential roles of viruses in regulating the biological processes of their hosts.

## Endogenous viral elements in mammalian genomes

Retroviruses are found in almost all mammals and other vertebrates, and approximately 8% of the human genome is composed of retroviruses in the form of endogenised proviruses.^29^, ^30^ Given that only about 1% of genomic DNA is made up of protein‐coding genes, the abundance of retrovirus sequences in the human genome is remarkable. Retrovirus sequences are conserved in humans and other primates, and therefore, the endogenisation of retroviruses is thought to have occurred millions of years ago.^31^ Some endogenous retroviruses have been shown to play beneficial roles in their hosts, including host evolution. For example, envelope genes from endogenous retroviruses contribute to the formation of the placenta during the fusion of syncytiotrophoblast cells in mammals.^32^, ^33^ In addition, endogenous retroviral elements are known to protect host cells from infection with exogenous retroviruses in some mammals.^33^, ^34^, ^35^

In addition to retroviruses, recent studies have shown that non‐retroviral viruses have also endogenised in many mammalian species.^36^, ^37^, ^38^ For example, Tomonaga's group, which is one of the research groups in our Neo‐virology research consortium, discovered that bornaviruses, a genus of non‐segmented, negative‐strand RNA virus, have been endogenised in the genomes of many mammals, including humans.^36^ Since bornaviruses do not encode reverse transcriptase and integrase genes, integration of bornavirus segments is believed to be mediated by long interspersed nuclear element‐1 (LINE‐1), which is a retrotransposon widely distributed in mammalian genomes.^39^ Tomonaga's group also showed that an endogenous bornavirus‐like element in the ground squirrel genome blocks infection and replication of extant endogenous bornavirus.^40^ Together, these findings indicate a potential role for endogenous non‐retroviral elements in antiviral defence. This group also performed an evolutionary phylogenetic analysis to elucidate the functions of endogenous bornavirus in mammalian genomes.^41^

Thus, it has become apparent that a large number of endogenous viral elements have accumulated in host genomes, more than previously expected. Therefore, it is important that we understand the significance of endogenous viral elements to the biological function and evolution of hosts.

## Diversity of viruses: discovery of novel viruses in nature

The first virus to be identified in humans was the yellow fever virus in 1901 after the discovery of tobacco mosaic virus in 1892 in plants and foot‐and‐mouth disease virus in 1898 in animals.^42^ Since then, new virus species that infect humans have been identified almost every year. Woolhouse *et al*.^43^ reviewed human viruses that had been described in the literature and recognised by the International Committee on Taxonomy of Viruses (ICTV), and drew a discovery curve for human viruses by plotting the cumulative number of species reported to infect humans; they showed that new species of human viruses have been discovered at a rate of three or four per year. Currently, there are approximately 263 viruses from 25 viral families that are known to be able to infect humans according to the latest ICTV report.^44^

In the last a few decades, emerging infectious diseases caused by newly identified viruses, such as Ebola virus,^5^, ^6^, ^7^, ^8^ SARS and MERS coronaviruses,^10^, ^11^, ^12^ human immunodeficiency virus (HIV),^9^ Nipah virus and Hendra virus,^45^, ^46^, ^47^, ^48^ have appeared in human society. Most emerging infectious diseases are zoonotic, caused by viruses that originate in wild animals, such as primates, rodents and bats^49^, ^50^, ^51^; in particular, bats have drawn attention because a recent comprehensive analysis of mammalian host–virus relationships indicated that bats have a significantly higher proportion of zoonotic viruses than all other mammalian orders.^52^ This analysis was part of a study supported by the United States Agency for International Development (USAID) Emerging Pandemic Threats PREDICT programme (https://ohi.vetmed.ucdavis.edu/programs-projects/predict-project), which was initiated in 2009, working with partners in over 30 countries on global surveillance of viruses to identify and monitor zoonotic pathogens. To date, the PREDICT programme has found over 1100 viruses in animals and humans, including a new Ebola virus and MERS‐ and SARS‐like coronaviruses. In 2018, the Global Virome Project (http://www.globalviromeproject.org) was launched, which aims to conduct viral surveillance on an even larger scale than the PREDICT programme. Those involved in this project estimate that ~1.67 million yet‐to‐be‐discovered viral species from key zoonotic viral families exist in mammal and avian hosts, and expect that 631 000–827 000 of these unknown viruses have zoonotic potential.^44^ In addition to viral surveillance in mammals and avian hosts, Zhang and Holmes's group recently conducted a screen for RNA viruses in diverse host species (more than 186 species other than mammalian and avian hosts) and identified about 200 vertebrate‐associated RNA viruses in fishes, reptiles and amphibians.^53^ They also found that vertebrate‐specific viral families or genera known to infect mammals and birds, including influenza viruses, filoviruses and hantaviruses, are also present in amphibians, reptiles and fish.^53^

In addition to the identification of new virus species in diverse hosts, new virus lifestyles have also been found in nature. Suzuki's group, which is one of the groups in our Neo‐virology research consortium, recently reported a new virus lifestyle exhibited by two RNA viruses: a double‐stranded (ds) RNA virus (yado‐nushi virus 1, YnV1) and a positive‐sense, single‐stranded [(+)ss] RNA virus (yado‐kari virus 1, YkV1) in a phytopathogenic fungus, *Rosellinia necatrix*.^54^ They found that the (+)ssRNA virus (YkV1), which does not have its own capsid protein, hijacks the capsid protein of the dsRNA virus (YnV1) to replicate.^55^

## Conclusion

Our world is made up of vastly different physical environments and the various organisms that have adapted to live in those environments. The complex interactions between the living and non‐living components of these environments are the basis of the global ecosystem. Various schemes have been proposed to classify living organisms: the one most often used currently defines all living organisms as archaea, bacteria or eukaryotes. Therefore, viruses are not considered living components of the global ecosystem. Given that approximately 10^31^ virus particles exist on Earth^56^, ^57^ and all of them are parasitic in living organisms, it is not hard to imagine how virus infection might affect the physiological functions of both hosts and the ecosystem. Although ‘traditional’ virology research tends to focus on pathogenic viruses that cause diseases in their hosts, the recent progress in next‐generation sequencing (NGS) technologies and data analyses has enabled us to discover a wide variety of new viruses, some of which do not cause diseases in their hosts. Some obstacles to comprehensive virome analyses remain, such as viral dark matter, which are sequences that originate during virus metagenomics but cannot be aligned to any reference sequences of viruses.^58^ Nonetheless, recent viral metagenomic studies using NGS technologies and bioinformatic analyses have identified a large number of viruses in environmental samples, including plants and oceans.^59^, ^60^ Characterisation of these newly identified viruses may provide new insight into the significance of viruses and virus‐mediated processes within global ecosystems.

## Conflict of interest

YK has received speaker's honoraria from Toyama Chemical and Astellas and grant support from Chugai Pharmaceuticals, Daiichi Sankyo Pharmaceutical, Toyama Chemical, Tauns Laboratories, Otsuka Pharmaceutical and Kyoritsu Seiyaku; and is a founder of FluGen.
